# Suspected Lamotrigine-Associated Haematological Adverse Reaction in the Context of Polypharmacy, Alcohol Use, HIV, and Recent Medication Changes

**DOI:** 10.7759/cureus.113682

**Published:** 2026-07-30

**Authors:** Tolulope Alabi, Anil Kumar

**Affiliations:** 1 Psychiatry, Priory Hospital, Roehampton, GBR; 2 Psychiatry, South London and Maudsley NHS Foundation Trust, London, GBR

**Keywords:** hiv psychiatry, lamotrigine-associated drug reaction, mood stabiliser, pancytopenia, psychiatric comorbidity

## Abstract

This is a case report of an adult male with well-controlled HIV infection who developed fever, malaise, myalgia and headache within 24-48 hours of commencing lamotrigine. Laboratory investigations demonstrated acute leukopenia, neutropenia and thrombocytopenia, accompanied by elevated inflammatory markers. Following withdrawal of lamotrigine, constitutional symptoms improved rapidly, while haematological and inflammatory markers demonstrated an overall trend towards recovery over subsequent days, although some laboratory abnormalities transiently persisted or worsened before normalising. This case highlights the importance of considering drug-induced haematological toxicity in patients presenting with fever and constitutional symptoms shortly after initiating lamotrigine, as prompt recognition and discontinuation may facilitate rapid recovery.

## Introduction

Lamotrigine (LTG) is a phenyltriazine anticonvulsant, licensed in the United Kingdom for monotherapy or combination therapy for bipolar disorder in adults and treatment of epilepsy [1,2]. Beyond its licensed indications, LTG is also used off-label in psychiatric practice as an augmentation or mood-stabilising strategy in selected patients with treatment-resistant or recurrent depressive disorders, particularly where affective instability is a prominent clinical feature, although the supporting evidence is less robust than for bipolar disorder [3]. Owing to its favorable efficacy and tolerability profile, particularly its effectiveness in preventing depressive relapse in bipolar disorder, LTG has become an important component of long-term mood stabilization [4].

Although LTG is generally well tolerated, its association with severe cutaneous adverse reactions, including Stevens-Johnson syndrome (SJS) and toxic epidermal necrolysis (TEN), is well recognized and reflected in prescribing guidance [1,2]. Less commonly recognized are its haematological adverse effects, including neutropenia, leukopenia, anaemia, thrombocytopenia, pancytopenia, aplastic anaemia and agranulocytosis, which are considered very rare (<1/10,000) [2].

Several case reports have described severe neutropenia and agranulocytosis associated with LTG, highlighting the importance of early recognition [5]. Beyond describing a rare adverse reaction, this report serves as an educational case illustrating the challenges of diagnosing drug-induced haematological toxicity in patients with multiple potential confounding factors, including medical comorbidity, recent medication changes and possible intercurrent infection. It emphasises the importance of careful clinical reasoning and temporal assessment when evaluating suspected adverse drug reactions.

## Case presentation

We report a case of an adult male, in his 30s, admitted informally for worsening emotional distress, low mood, poor sleep, increased alcohol use, and concerns regarding his ability to cope with stressors.

He had started seeing an external psychiatrist three years before, due to significant anxiety (regarding oral discomfort) which escalated into panic-like episodes, irritability, sleep problems, and functional impairment. The psychiatrist made a diagnosis of Generalised Anxiety Disorder and treated this with pregabalin, diazepam, flupentixol/melitracen, and later escitalopram. His symptoms improved after nine to 12 months, and medications were discontinued. However, following recurrence of symptoms, the psychiatrist commenced desvenlafaxine and lemborexant, which proved effective.

In terms of physical health, he was diagnosed with HIV in his early 20s and had been on darunavir/cobicistat 800mg/150mg and emtricitabine/tenofovir alafenamide 200mg/10mg, with viral load undetectable for 15 years. His reported baseline creatinine had historically been high with low estimated glomerular filtration rate (eGFR), although more recent measurements had shown an upward trend. We attributed this largely to his use of creatine supplements, as we observed a temporal relationship between elevated creatinine and use of the supplements on admission (see Table 1). Dehydration from alcohol binges was also a possibility, as when drinking socially, he reported consuming alcohol (which might equate to a bottle of wine and a few beers) on Thursdays, Fridays, and Saturdays, often to the point of intoxication.

**Table 1:** Creatinine and calculated eGFR trend on admission eGFR: Estimated Glomerular Filtration rate, LTG: lamotrigine

Parameter	Reference range	Baseline/admission	4 days after LTG initiation (day 12 of admission)	4 days after LTG discontinuation (day 16 of admission)	8 days after LTG discontinuation (day 20 of admission)
Creatinine	59-104 umol/L	123 umol/L	127 umol/L	114 umol/L	115 umol/L
eGFR/1.72m2	-	64 mL/min	62 mL/min	70 mL/min	70 mL/min

On review by our team, the diagnostic formulation included: 6A71.1 Recurrent depressive disorder, current episode moderate, without psychotic symptoms; 6A80.0 Prominent anxiety symptoms in mood episodes; 6B40 Post-traumatic stress disorder (PTSD) or complex PTSD.

At admission, he had blood tests done and was prescribed his usual medications (agomelatine 25mg once daily, emtricitabine/tenofovir alafenamide 200mg/10mg once daily, finasteride 1mg once daily, darunavir/cobicistat 800mg/150mg once daily, and desvenlafaxine 50mg once daily).

Despite an initial response to desvenlafaxine, the patient experienced a recurrence of oral discomfort together with affective instability characterised by fluctuating mood, irritability, and difficulty coping with psychosocial stressors. Following psychiatric review, we elected to optimise antidepressant therapy by switching desvenlafaxine to duloxetine while introducing LTG 25mg once daily as an off-label mood-stabilising augmentation strategy. The decision reflected the clinical impression that emotional dysregulation was contributing significantly to the patient's presentation.

The following day, the patient reported fever, generalized aches and pains, poor sleep, feeling “hot and cold”, sweating, weakness, headache, nausea, lethargy, all of which he reported had started the night before. Physical examination during the initial 48 hours showed elevated temperatures ranging from 38.5°C to 39.1°C and tachycardia, with preserved oxygen saturation. No rash, no mucosal blistering, and no respiratory compromise were observed.

The differential diagnoses at this stage included antidepressant discontinuation syndrome, viral illness, and an early adverse drug reaction. Potential drug interactions were reviewed. Although interaction resources recommend caution when LTG and duloxetine are co-prescribed, no established pharmacokinetic interaction was identified that would be expected to account for the observed clinical presentation.

Repeat blood tests four days after commencing LTG showed a decrease in white blood cell count (WBC) from a baseline level of 5.8 x 10*9/L to 2.2 x 10*9/L; neutrophil count decreased from 3.0 x 10*9/L to 0.8 x 10*9/L, and platelet count dropped from 233 x 10*9/L to 126 x 10*9/L (see Table 2).

**Table 2:** Blood results comparison table ALT: alanine aminotransferase; CRP: C-reactive protein; ESR: erythrocyte sedimentation rate; LTG: lamotrigine; RBC: red blood cells; WBC: white blood cell count; WCC: white cell count.

Parameter	Reference range	Baseline/admission	4 days after LTG initiation (day 12 of admission)	4 days after LTG discontinuation (day 16 of admission)	8 days after LTG discontinuation (day 20 of admission)
WBC	4.0-10.0 x 10*9/L	5.8 x 10*9/L	2.2 x 10*9/L	6.6 x 10*9/L	5.1 x 10*9/L
Neutrophils	2.0-7.0 x 10*9/L	3.0 x 10*9/L	0.8 x 10*9/L	1.0 x 10*9/L	2.2 x 10*9/L
Platelets	150-410 x 10*9/L	233 x 10*9/L	126 x 10*9/L	108 x 10*9/L	332 x 10*9/L
RBC	4.50-5.50 x 10*12/L	4.55 x 10*12/L	4.65 x 10*12/L	4.37 x 10*12/L	4.36 x 10*12/L
Lymphocytes	1.0-3.0 x 10*9/L	2.0 x 10*9/L	1.0 x 10*9/L	4.89 x 10*9/L	2.2 x 10*9/L
ESR	0-10 mm/h	10 mm/h	7 mm/h	22 mm/h	31 mm/h
ALT	1-41 U/L	31 U/L	35 U/L	71 U/L	73 U/L
Ferritin	30-400 ug/L	341 ug/L	-	447 ug/L	319.0 ug/L
CRP	<5 mg/L	1.9 mg/L	9.5 mg/L	15.9 mg/L	1.9 mg/L

Both duloxetine and LTG were considered as they had been commenced concurrently before onset of symptoms. However, duloxetine is not commonly associated with acute leukopenia, severe neutropenia and thrombocytopenia developing within days of treatment initiation. In contrast, LTG has recognised, albeit rare, haematological adverse effects described in the product information and published case reports. In view of the close temporal relationship between LTG initiation, symptom onset, and the evolving cytopenias, together with the potential severity of LTG-associated blood dyscrasias, LTG was considered the more likely causative agent and was discontinued as a precautionary measure. At that stage, there were no localising features of infection.

Within a day of discontinuation, his symptoms started to resolve, and he had blood tests repeated to monitor improvement.

Four days after discontinuation WBC increased to 6.6 x 10*9/L; neutrophil count, although low, went up to 1.0 x 10*9/L and platelet was lower at 108 x 10*9/L. Bloods done also showed low red blood cell count (RBC) 4.37 x 10*12/L, raised lymphocytes 4.89 x 10*9/L, raised erythrocyte sedimentation rate (ESR) 22, high alanine aminotransferase (ALT) 71 U/L, elevated ferritin 447 ug/L, and raised C-reactive protein (CRP) 15.9 mg/L (see Table 2).

Five days after discontinuation, symptoms had completely resolved, and he reported feeling more like himself. However, he also noted a sore throat with no other associated symptoms. Due to the associated laboratory findings, including raised ALT and inflammatory markers, serology test was done, which detected Epstein-Barr virus viral capsid antigen (EBV VCA) IgG but not IgM. The sore throat was managed with painkillers and lozenges.

By the eighth day after discontinuation, WBC was 5.1 x 10*9/L, neutrophil count 2.2 x 10*9/L, and platelets 332 x 10*9/L. Previously elevated lymphocyte (2.2 x 10*9/L), ferritin (319.0 ug/L), and CRP (1.9 mg/L) were also within the normal range. However, ESR rose to 31, and RBC remained low at 4.36 x 10*12/L (see Table 2). The presence of a stye (hordeolum) at the time of this blood test may have been responsible for elevated ESR.

The patient was discharged after three weeks on admission (nine days after discontinuing LTG) as previously planned. He was advised to have repeat blood tests done on an outpatient basis, and this was highlighted in the discharge summary.

## Discussion

Several mechanisms have been proposed to explain LTG-associated blood dyscrasias. These include inhibition of dihydrofolate reductase, direct bone marrow toxicity, and immune-mediated mechanisms associated with drug hypersensitivity reactions [2,5,6]. Although our patient did not develop the characteristic rash or eosinophilia, immune-mediated mechanisms remain one of several proposed explanations for LTG-associated haematological toxicity.

Multiple published case reports have described severe neutropenia, agranulocytosis, or pancytopenia associated with LTG [5,7,8]. However, most occurred after several days of therapy, often during dose escalation or following higher initial doses [5,7,8]. In contrast, our patient developed systemic symptoms within 24 hours and objective haematological abnormalities within 96 hours despite initiation at the recommended low dose of 25mg daily. This unusually early presentation highlights that clinically significant haematological toxicity may occur even when current prescribing recommendations and slow dose titration are followed. In all cases, including ours, recovery started soon after discontinuation of LTG.

LTG undergoes hepatic glucuronidation primarily through UGT1A4, and alterations in drug clearance or pharmacokinetic interactions may theoretically increase systemic drug exposure in susceptible individuals [9]. Several published cases have involved concomitant medications capable of altering LTG metabolism or increasing susceptibility to adverse drug reactions, suggesting that polypharmacy may be an important predisposing factor [5,10-12]. Unlike several previously reported cases, our patient was not receiving medications with well-established clinically significant pharmacokinetic interactions with LTG (see Table 3). 

**Table 3:** Comparison with previously published cases. Compared with previously published reports, the present case demonstrated unusually early constitutional symptom onset and rapid development of cytopenias despite initiation at the recommended lamotrigine (LTG) starting dose and in the absence of cutaneous manifestations.

Study	Patient	Indication	Initial dose of LTG/ titration of LTG/co-administered medication	Time to onset of haematological abnormality	Haematological abnormality	Clinical features	Management	Outcome
de Camargo & Bode (1999) [7]	11-year-old female	Epilepsy	50mg once daily	Two weeks	Agranulocytosis	Maculopapular rash, slight pruritus, some abdominal discomfort.	LTG stopped.	1. Clinical symptoms resolved in five days. 2. After 10 days, leucocyte count improved, increasing during the following week. 3. Liver enzyme concentrations remained slightly raised.
Chang et al. (2006) [11]	48-year-old female	Bipolar disorder	50mg once daily, increased to 100mg once daily on day 4 (LTG was added to a regimen of valproic acid and venlafaxine)	Seven days	Leucopenia Thrombocytopenia	Generalized maculopapular rash, pancytopenia, pneumonitis, hepatitis	LTG and valproic acid stopped. Administration of oral antihistamine and corticosteroid.	Recovery following management.
Yasui-Furukori et al. (2014) [8]	19-year-old female	Bipolar disorder	25mg once daily, increased to 50mg once daily on day 10	Ten to 13 days	Neutropenia	Stevens-Johnson syndrome, which had developed within 10 days	LTG stopped. Steroid pulse therapy.	Firstly, her symptoms and dermatological manifestations revealed a slight improvement. White blood cell count initially dropped further, before starting to improve from two days after commencement of steroid pulse therapy (five days after stopping LTG)
Salem & El-Bardissy (2021) [5]	29-year-old female	Epilepsy	50mg twice daily, increased to 150mg once daily on day 3, then reduced to 100mg once daily on day 7, before being increased back to 150mg daily on day 14 (LTG was initially co-administered with phenytoin. At the time of initial increase to 150mg, phenytoin had been stopped before being re-introduced, briefly for 5 days, after stopping LTG.)	Two weeks	Severe neutropenia	Fever without rash	LTG stopped. Administered multiple antibiotics. Haematology team involvement and Filgrastim commencement due to continued drop in White blood cell count and neutrophils (reached 0.0 x10*9/L) five days after stopping LTG. Recommenced phenytoin changed to levetiracetam, five days after stopping LTG.	1. Fever initially subsided immediately after LTG discontinuation but spiked five days later. 2. Fever resolved within a day and hematology parameters started to improve two days after commencement of Filgrastim.
Present case	Male in 30s	Mood stabilization	25mg/day (recommended starting dose)	Constitutional symptoms: 24 hours. Cytopenias detected: 96 hours	Leukopenia, severe neutropenia, and thrombocytopenia	Fever, malaise, myalgia, headache	LTG stopped	Clinical symptoms recovery starts immediately after. Improvements in hematology parameters noted four days after stopping LTG.

Recent antidepressant switching also warranted careful consideration. Discontinuation symptoms following abrupt cessation of desvenlafaxine may include headache, sweating, dizziness, nausea, anxiety, irritability, and flu-like symptoms, potentially mimicking an acute adverse drug reaction [13]. However, antidepressant withdrawal would not be expected to produce severe neutropenia, leukopenia, and thrombocytopenia, making it an insufficient explanation for the overall clinical presentation. 

The concurrent initiation of duloxetine was considered when assessing causality. As duloxetine replaced desvenlafaxine, another serotonin-norepinephrine reuptake inhibitor, the risk of antidepressant discontinuation syndrome was likely reduced [13]. Furthermore, although duloxetine may cause non-specific adverse effects, including headache, nausea, sweating, and dizziness, it is not commonly associated with the acute onset of combined leukopenia, severe neutropenia, and thrombocytopenia observed in this patient [14,15]. In contrast, these haematological abnormalities have been reported, albeit rarely, in association with LTG. Accordingly, LTG was considered the more probable causative agent, although a contributory role of duloxetine cannot be entirely excluded.

Similarly, although alcohol misuse can contribute to transient bone marrow suppression [16], the patient's baseline blood counts were entirely normal on admission despite his established drinking pattern, making alcohol alone less likely to explain the abrupt deterioration observed immediately after LTG initiation.

Although clinically significant pharmacokinetic interactions between LTG and the patient's concomitant medications are not well established, the combination of polypharmacy, mild renal impairment, well-controlled HIV infection, recent antidepressant switching and constitutional symptoms created a complex clinical picture in which several competing explanations for the patient's presentation were plausible.

An infectious process remained an important differential diagnosis because the patient initially presented with fever, constitutional symptoms, and raised inflammatory markers. The sore throat, which developed a few days after discontinuing LTG, occurred during resolution of the initial constitutional symptoms. Given the new upper respiratory symptom, associated lymphocytosis, persistent thrombocytopenia, elevated inflammatory markers, and a background history of well-controlled HIV infection, Epstein-Barr virus serology was undertaken to evaluate for acute infectious mononucleosis. Epstein-Barr virus serology demonstrated viral capsid antigen IgG positivity without IgM positivity, supporting previous rather than acute infection. While an intercurrent infection cannot be completely excluded, the chronology of symptom onset, laboratory findings and clinical recovery was considered more consistent with a suspected LTG-associated adverse drug reaction than with infection as the primary cause of the presentation. It is also plausible that the transient leukopenia may have increased susceptibility to a subsequent minor infection, although this cannot be established with certainty.

HIV itself may cause cytopenias through chronic immune activation, opportunistic infection, or direct bone marrow effects [17]. However, HIV-related cytopenias generally occur in association with uncontrolled viraemia, opportunistic infection, or chronic marrow dysfunction. Our patient had maintained virological suppression for approximately 15 years and demonstrated normal baseline blood counts, making HIV an unlikely primary explanation.

Several features support a causal association between LTG and the observed haematological abnormalities. These include the close temporal relationship between treatment initiation and symptom onset, objective laboratory evidence of leukopenia, severe neutropenia and thrombocytopenia, rapid clinical improvement following LTG withdrawal, and consistency with previously published reports of LTG-associated haematological toxicity. Alternative explanations, including concurrent duloxetine initiation, antidepressant discontinuation syndrome, HIV-related cytopenia, alcohol misuse and an intercurrent infection, were carefully considered but were judged less likely based on the chronology of events, available investigations and the subsequent clinical course. Rechallenge was neither clinically appropriate nor ethically justified because of the potential severity of the reaction. Application of the original Naranjo Adverse Drug Reaction Probability Scale yielded a score of 5, consistent with a probable adverse drug reaction (see Table 4). However, the Naranjo algorithm has recognised limitations in the assessment of rare idiosyncratic reactions, particularly where rechallenge is contraindicated [18].

**Table 4:** Adverse Drug Reaction Probability Scale Naranjo Adverse Drug Reaction Probability Scale applied to the present case, adapted from Naranjo et al. [18]. A total score of 5 is consistent with a probable adverse drug reaction.

Question	Response	Score	Justification
1. Are there previous conclusive reports on this reaction?	Yes	+1	Multiple published case reports of lamotrigine-associated neutropenia, agranulocytosis, and pancytopenia.
2. Did the adverse event appear after the suspected drug was administered?	Yes	+2	Symptoms developed within 24–48 hours of starting lamotrigine; cytopenias were detected on day 4.
3. Did the adverse reaction improve when the drug was discontinued or a specific antagonist was administered?	Yes	+1	Constitutional symptoms improved within 24 hours, and blood counts subsequently recovered.
4. Did the adverse reaction reappear when the drug was readministered?	Not done	0	Rechallenge was not attempted because of safety concerns.
5. Are there alternative causes (other than the drug) that could on their own have caused the reaction?	Do not know	0	Alternative causes, including infection, concurrent duloxetine initiation, HIV, alcohol misuse and antidepressant discontinuation syndrome, were considered. Although each was judged less likely than lamotrigine based on the chronology, laboratory findings and clinical course, none could be confidently excluded.
6. Did the reaction reappear when a placebo was given?	Not applicable	0	No placebo challenge.
7. Was the drug detected in the blood (or other fluids) in concentrations known to be toxic?	Not done	0	Lamotrigine serum concentrations were not measured.
8. Was the reaction more severe when the dose was increased, or less severe when the dose was decreased?	Not assessable	0	Dose was not escalated before discontinuation.
9. Did the patient have a similar reaction to the same or similar drugs in any previous exposure?	No	0	No previous exposure or similar reaction documented.
10. Was the adverse event confirmed by any objective evidence?	Yes	+1	Laboratory investigations documented leukopenia, neutropenia, and thrombocytopenia.

Although causality cannot be established definitively in a single case with multiple confounding factors, the overall clinical evidence supports LTG as the most likely cause of the patient's presentation despite the presence of competing alternative explanations.

## Conclusions

This case report examined probable LTG-induced adverse clinical and haematological effects in a patient being treated for mood instability. Several concurrent clinical factors, including well-controlled HIV infection, a history of alcohol misuse, recent antidepressant changes, and the possibility of an intercurrent viral or inflammatory process, were considered during the diagnostic assessment. Although each may have contributed to the overall clinical picture, none was considered sufficient to independently explain the abrupt onset of fever together with leukopenia, severe neutropenia and thrombocytopenia shortly after LTG initiation. 

Ultimately, the case illustrates the challenges of diagnosing rare adverse drug reactions in patients with multiple potential confounding factors. Careful consideration of the temporal relationship between medication exposure, clinical features and serial laboratory findings is essential when assessing causality. Clinicians should maintain a high index of suspicion for LTG-associated haematological toxicity in patients who develop fever or constitutional symptoms shortly after treatment initiation, even in the absence of rash or other features of drug hypersensitivity. While routine serial full blood count monitoring is not warranted for all patients commencing LTG, prompt repeat blood testing and early review of the ongoing need for LTG should be considered in symptomatic patients to facilitate timely recognition and management of this rare but potentially serious adverse reaction.
